# Expression of RSPH6A in the first wave of rat spermatogenesis and oxidative stress conditions: Attenuation by melatonin

**DOI:** 10.1002/rmb2.12542

**Published:** 2023-10-02

**Authors:** Maria Zelinda Romano, Mariem Ben Rhouma, Imed Messaoudi, Francesco Aniello, Sergio Minucci, Massimo Venditti

**Affiliations:** ^1^ Dipartimento di Medicina Sperimentale, Sez. Fisiologia Umana e Funzioni Biologiche Integrate “F. Bottazzi” Università degli Studi della Campania “Luigi Vanvitelli” Napoli Italy; ^2^ Laboratoire LR11ES41 Génétique Biodiversité et Valorisation des Bio‐Ressourcés Institut Supérieur de Biotechnologie de Monastir, Université de Monastir Monastir Tunisia; ^3^ Dipartimento di Biologia Università di Napoli “Federico II” Napoli Italy

**Keywords:** cadmium, melatonin, rat testis, RSPH6A, spermatozoa

## Abstract

**Purpose:**

Here, we report, for the first time, the temporal expression and localization of axonemal radial spoke head homolog A (RSPH6A) protein during the first wave of rat spermatogenesis and in oxidative stress conditions.

**Methods:**

For the developmental study, testes were collected from rats at different developmental stages (7, 14, 21, 28, 35, 42, and 60 postnatal days); for *in vivo* *treatment*, 24 rats were treated with cadmium and/or melatonin. From each sample, western blot (WB) and immunofluorescence (IF) analyses for RSPH6A were performed.

**Results:**

RSPH6A expression starts at 21 PND alongside the appearance of I spermatocytes (SPC) with a significant increase up to 60 PND. Data were confirmed by IF analysis, showing that RPSH6A expression is restricted to I and II SPC, spermatids, and mature sperm. *In vivo* experiments showed that the expression and localization of RSPH6A in the testis and epididymal spermatozoa of adult rats treated with cadmium were impaired. Interestingly, melatonin (an antioxidant), given together with Cd, can counteract its damaging effects.

**Conclusions:**

All combined data confirm that RSPH6A contributes to the onset of fertility by acting on sperm motility, raising the possibility of using RSPH6A as a marker for normal fertility in the general population.

## Graphical abstract

Here is reported, for the first time, the temporal expression and localization of RSPH6A protein during the first wave of rat spermatogenesis. Its expression starts at 21 PND alongside the appearance of ISPC and increases up to 60 PND. The expression and localization of RSPH6A in the testis and epididymal spermatozoa of adult rats treated with cadmium were impaired. Melatonin, given together with Cd, can counteract its damaging effects.

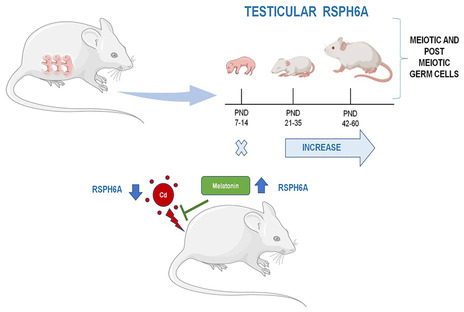

## 1 INTRODUCTION

Radial spoke proteins connect the central pair of microtubules and the outer microtubules in each axoneme and are essential for its distinctive curvature pattern and motility.^1^ RSPH6A (radial spoke head 6 homolog A), a component of the flagellar axoneme,^2^, ^3^ is a testis‐specific protein and, due to its specific localization in the tail of the mature spermatozoa (SPZ), is involved in flagellar motility.^4^

RSPH6A mRNA is exclusively expressed in testicular germ cells (GC) during meiosis, and the protein is associated with flagellar cytoskeletal structures in mature mouse sperm^2^ and in human testes and sperm flagella.^5^, ^6^

In addition, in our previous study, we found that RSPH6A protein expression and localization were compromised in the SPZ of myotonic dystrophy type 1 (DM1) patients, causing impaired motility and fertility,^6^ producing the fertility defects observed in DM1 patients.^6^, ^7^ Furthermore, our recent study showed that in human SPZ samples, cadmium (Cd) treatment, via producing oxidative stress, reduced RSPH6A protein levels and consequently affect SPZ motility; this effect was counteracted by melatonin (Mlt).^8^

A recent report showed that asthenospermia was present in 50.5% of infertile males examined^9^ since an SPZ efficient motility is necessary for their passage through the female reproductive tract.^10^, ^11^, ^12^ Decreased sperm motility significantly reduces SPZ ability both to reach the fertilization site and to penetrate the oocyte, thereby reducing male fertility; therefore, their motility is essential for the success of fertilization.^13^, ^14^

It is known that lifestyle and exposure to environmental pollutants, in addition to genetic and hormonal causes, are the most frequent etiologies of infertility.^15^ Among the environmental substances directly involved in growing infertility, Cd is a heavy metal with a very long half‐life^16^ that acts as an endocrine disruptor^17^, ^18^ and induces oxidative stress.^19^, ^20^, ^21^, ^22^ Cd deleterious effects on health and on the fertility rate are widely studied,^23^ since Cd impairs spermatogenesis,^18^, ^21^, ^24^, ^25^, ^26^, ^27^ finally leading to a reduction in sperm quantity and quality, specifically by reducing SPZ motility.^28^, ^29^

Because Cd exposure is an inevitable phenomenon, numerous studies have concentrated on research to identify the precise molecular targets of Cd effects and, therefore, find potential ameliorative or suppressive molecules that could be utilized in new therapeutic advances. Of such protective molecules, melatonin (Mlt) is one of the most studied since it is known to have antiapoptotic and antioxidant effects.^30^ Our previous study indicated the ability of Mlt to counteract/protect the morphology and physiology of the rat testis from the detrimental effects of Cd.^18^, ^21^

Considering that the motility of SPZ is a crucial factor for the success of fertilization and keeping in mind that RSPH6A is just involved in their motility, herein, for the first time, we evaluated the expression of RSPH6A protein during postnatal development, other than the effect of in vivo Cd‐Mlt treatments on its protein level in rat testis and SPZ.

## 2 MATERIALS AND METHODS

### 2.1 Animals, treatments, and sample collection

#### 2.1.1 Development study

Male Wistar rats were kept under specific conditions (12:12 h light/dark) and received standard food and water ad libitum. Animals at different development stages (7, 14, 21, 28, 35, 42 and 60 postnatal days, PND) (*n* = 3 per stage) were anesthetized by intraperitoneal injection of ketamine (100 mg/kg i.p.) and then sacrificed.

Testes were removed, and for each rat, right testis was fixed in Bouin's solution for histological studies, while the left was stored at –80°C for biomolecular studies.

#### 2.1.2 In vivo treatment

Twenty‐four Wistar male rats, aged 2 months and weighing 225 ± 36 g, were kept in individual stainless‐steel cages under controlled conditions of light (12:12 h light/dark), temperature (22 ± 2°C) and humidity (55 ± 20%). Food and water were given *ad libitum*. Rats were divided into four groups (*n* = each); 1) control (Ctrl); 2) Cd‐treated (50 mg CdCl_2_/L in drinking water; Sigma‐Aldrich); 3) Mlt‐treated (3 mg/L in drinking water; Sigma‐Aldrich); 4) Cd + Mlt‐treated (50 mg CdCl_2_/L + 3 mg Mlt/L in drinking water). The concentration of Cd and Mlt was chosen according to the literature.^31^, ^32^ After 40 days, rats were sacrificed, and testes were sampled as above‐described. Epididymides were dissected and minced in PBS (pH 7.4) to allow SPZ to flow from the ducts. Then, the fluid samples were filtered and examined under a light microscope to exclude contamination by other cell types. Next, aliquots were spotted and air‐dried on slides and stored at −20°C; the remaining samples were centrifuged at 1000× g for 15 min at 4°C and stored at −80°C for molecular analysis.

The procedure was approved by the Ethics Committee for Research in Life Science and Health of the Higher Institute of Biotechnology of Monastir (CER‐SVS/ISBM – protocol 022/2020) and was carried out according to the UNESCO Recommendation Concerning Science and Scientific Research (1974, 2017).

### 2.2 Histology

The fixed testes were dehydrated in increasing ethanol concentrations before paraffin embedding. Five micrometres thick paraffin sections were stained with Hematoxylin/Eosin for histological evaluation.

### 2.3 Western blot (WB) analysis

Proteins were extracted from the testis and SPZ in RIPA lysis buffer [(#TCL131; HiMedia Laboratories GmbH) supplemented with protease inhibitors mix (#39102.01; SERVA Electrophoresis)]. The homogenized samples were sonicated 3 times (20 Hz for 20 s each), placed on ice for 30 min, centrifuged at 10000× g for 30 min at 4°C and the supernatants collected.^33^

Forty micrograms of the protein extracts were separated by 9% SDS‐PAGE and then transferred to Hybond‐P PVDF membranes (#GE10600023; Amersham Pharmacia Biotech) at 280 mA for 2.5 h at 4°C. Filters were blocked with 5% powdered milk in TBST (10 mM Tris–HCl pH 7.6, 150 mM NaCl, containing 0.25% Tween – 20) for 3 h at RT. Then, they were incubated with primary antibodies anti‐RSPH6A (80 kDa, 1:1000; #HPA045382, Sigma–Aldrich) and anti‐α‐Tubulin (52 kDa, 1:5000; #E‐AB‐20036, Elabscience Biotechnology).

After incubation at 4°C overnight, the filters were washed three times in TBST and incubated with peroxidase‐conjugated secondary antibody anti‐mouse IgG (1:5000; #AP130P; Sigma‐Aldrich) for the mouse anti‐α‐Tubulin or anti‐rabbit IgG (1:3000; #AP307P; Sigma‐Aldrich) secondary antibody for the anti‐RSPH6A, for 1 h at RT.

Filters were washed again in TBST three times. The immunocomplexes were detected using the enhanced chemiluminescence (ECL) – WB detection system. ImageJ software (version 1.53 g; NIH) was used to analyze all bands. Each WB was performed in triplicate.

### 2.4 Immunofluorescence (IF) analysis

For IF staining, testis sections, and sperms were permeabilized with PBS pH 7.4 containing 0.1% Triton‐X‐100 for 30 min. Antigen retrieval was performed by putting slides in a pressure cooker for 3 min in 0.01 M citrate buffer (pH 6.0). Non‐specific binding sites were blocked with PBS containing 5% BSA and normal goat serum diluted 1:5.^34^ Sections were incubated with primary antibodies anti‐RSPH6A (1:100); anti‐SYCP3 (1:100; #sc‐74 569; Santa Cruz Biotechnology), and anti‐α‐Tubulin (1:100) at 4°C. After three washes in PBS, slides were incubated for 1 h with PNA lectin (#L32458; Thermo Fisher Scientific) diluted at 1:50, and the appropriate secondary antibody [goat anti‐rabbit Alexa Fluor 488, (#A32731 Thermo Fisher Scientific); goat anti‐mouse CF™ 568 (#SAB4600082; Sigma–Aldrich)] both diluted to 1:500 in the blocking mixture for 1 h at RT. Nuclei were stained with Vectashield + DAPI and slides were observed and captured with the optical microscope (Leica DM 5000 B + CTR 5000) with a UV lamp and saved with IM 1000 software. Each IF was performed in triplicate.

### 2.5 Statistical analysis

Data are reported as mean ± SEM. Differences between the groups were considered statistically significant at *p* < 0.05. Analyses were performed using one‐way ANOVA; Tukey's post hoc *t*‐test was applied when appropriate with Prism 5.0, GraphPad.

## 3 RESULTS

### 3.1 RSPH6A protein expression and localization during development and in adult testis

**FIGURE 1:**
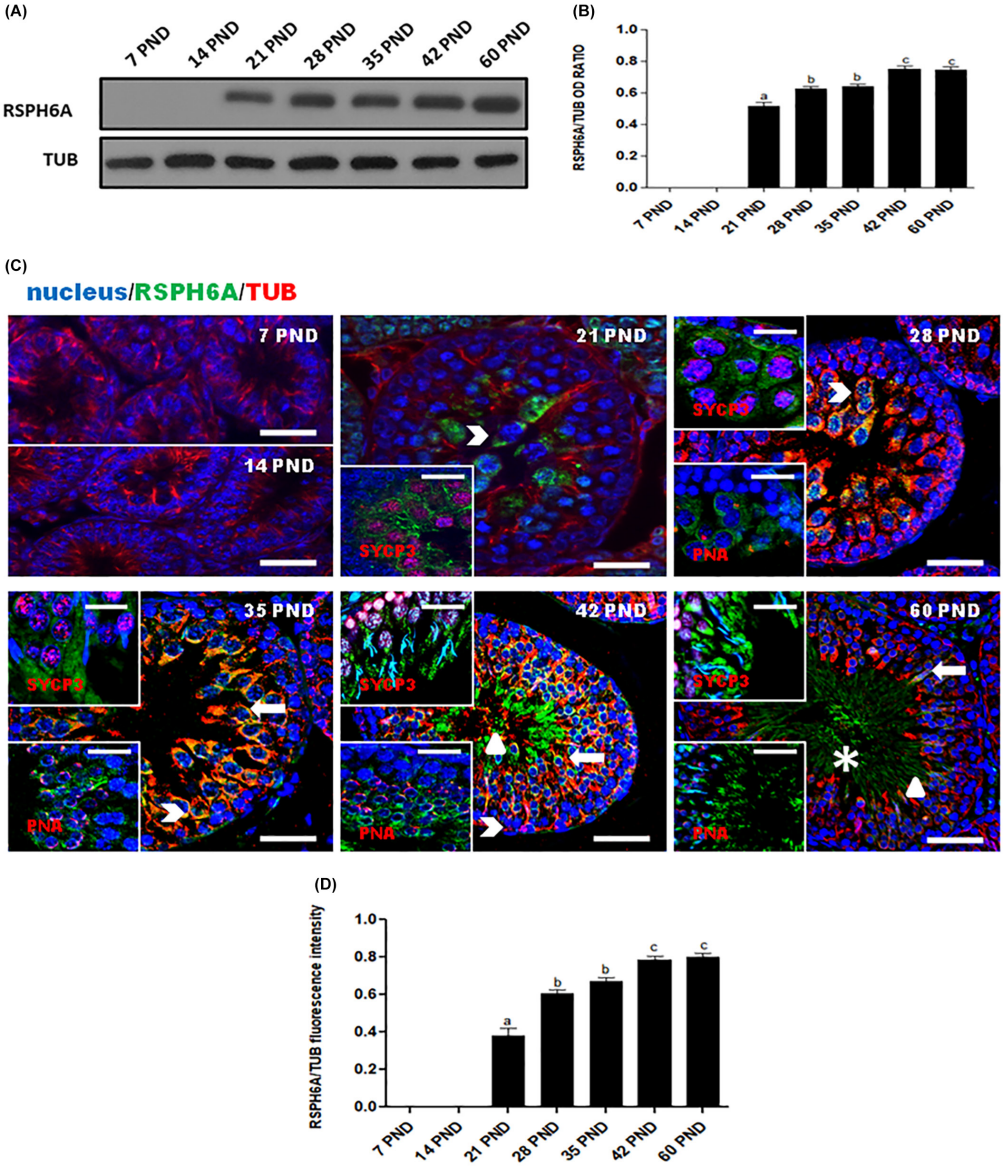
(A) WB and (B) relative histogram of RSPH6A in testis during developmental stages. Data were normalized with α‐Tubulin and reported as OD ratio. a vs. b *p* < 0.01; b vs. c *p* < 0.05. (C) IF analysis of RSPH6A (green) and α‐Tubulin (red) in testis during developmental stages. Cell nuclei were marked with DAPI (blue). The upper insets represent SYCP3 (red) and the lower insets represent PNA lectin (red). Arrowhead: SPC; thick arrow: RSpt; triangle: ESpt; asterisk: SPZ. Scale bars represent 20 μm and 10 μm in the insets. (D) Histogram showing the quantification of RSPH6A fluorescence signal intensity. a vs. b *p* < 0.001; b vs. c *p* < 0.01. All values are expressed as means ± SEM.

To assess the expression level of RSPH6A protein in the rat testis during postnatal development, we performed a WB analysis (Figure 1A). Its expression occurs from 21 PND at the onset of meiotic spermatocytes (SPC; Figure 1A) and significantly increases at 28 and 35 PND (*p* < 0.01), up to 42 and 60 PND (*p* < 0.01; Figure 1B). To clarify the protein localization, we performed an IF analysis for RSPH6A, together with tubulin, SYCP3 (a meiotic marker), and PNA lectin, which highlights the acrosome (Figure 1C). The results confirmed the WB findings; in fact, the appearance of the protein signal was observed starting at 21 PND in meiotic SPC, which were distinguished by the SYCP3 nuclear staining (arrowhead – Figure 1C) and, in the subsequent cell stages, marked by PNA lectin staining, the signal was also observed in round spermatids (RSpt; thick arrow – Figure 1C), elongated spermatids (ESpt; triangle – Figure 1C) and, lastly, in luminal SPZ (asterisk – Figure 1C). Analysis of the fluorescence intensity showed a comparable pattern, statistically significant, as observed for the protein level (Figure 1D).

### 3.2 Testicular morphology of Cd and Mlt‐treated testis

**FIGURE 2:**
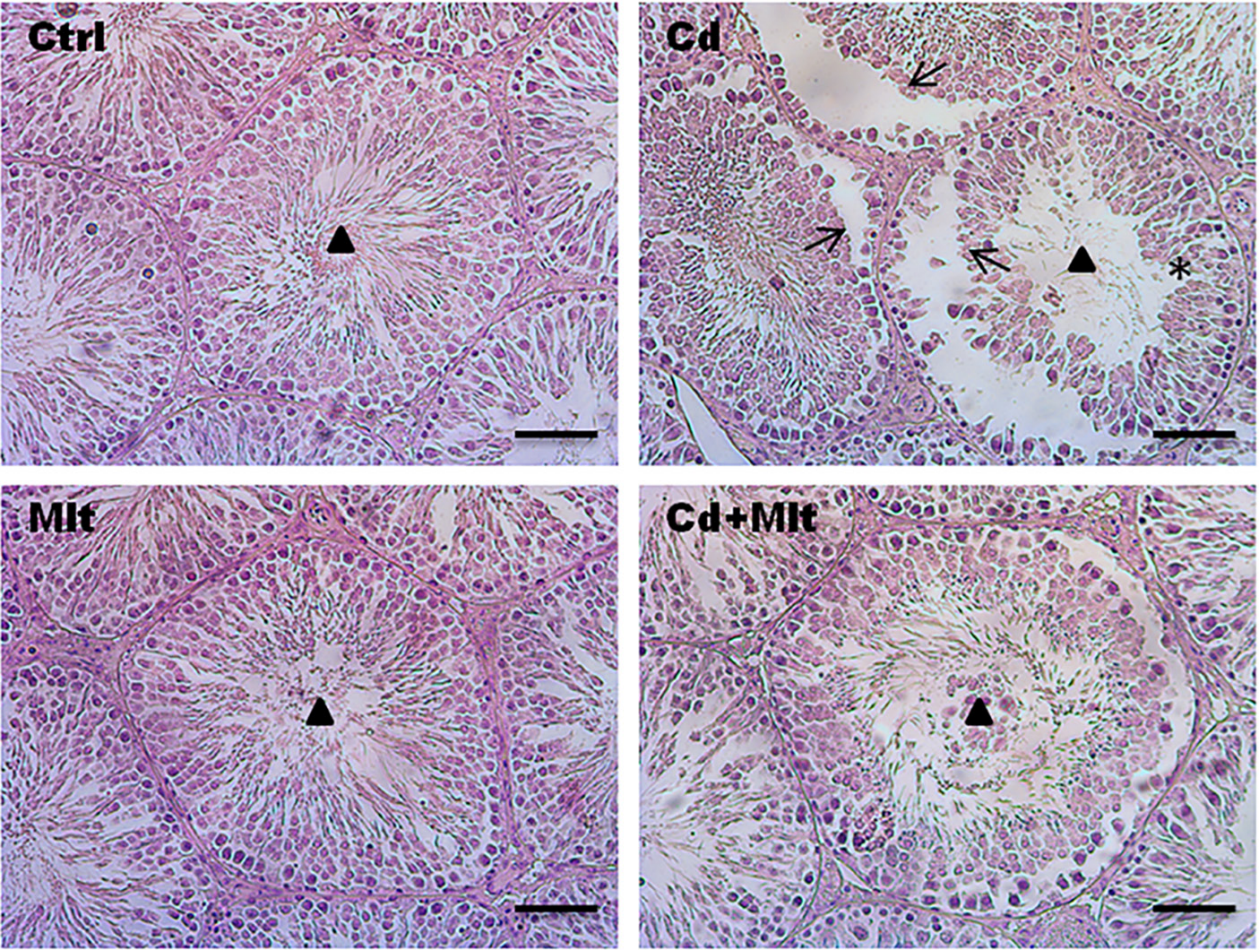
Hematoxylin/Eosin stain of Cd and Mlt‐treated testis. Morphological evaluation of testes treated with Cd and/or Mlt. Thin arrow: GC desquamation; Triangle: lumen; Asterisk: space between GC Scale bars represent 20 μm.

To evaluate the histological characteristics of the testis of Cd‐ and Mlt‐treated rats, Hematoxylin–eosin staining was performed (Figure 2). Control and Mlt‐treated rats presented an intact seminiferous epithelium and GC in all the different stages of differentiation and with lumens filled with mature SPZ (triangle). The Cd‐treated group showed non‐homogeneous seminiferous tubules, characterized by desquamation (thin arrow) and space between GC (asterisk). In the Cd + Mlt treated group, due to the counteractive action of Mlt, improvement in tubules and epithelium morphology was observed.

### 3.3 RSPH6A expression and localization in Cd and Mlt‐treated testis

**FIGURE 3:**
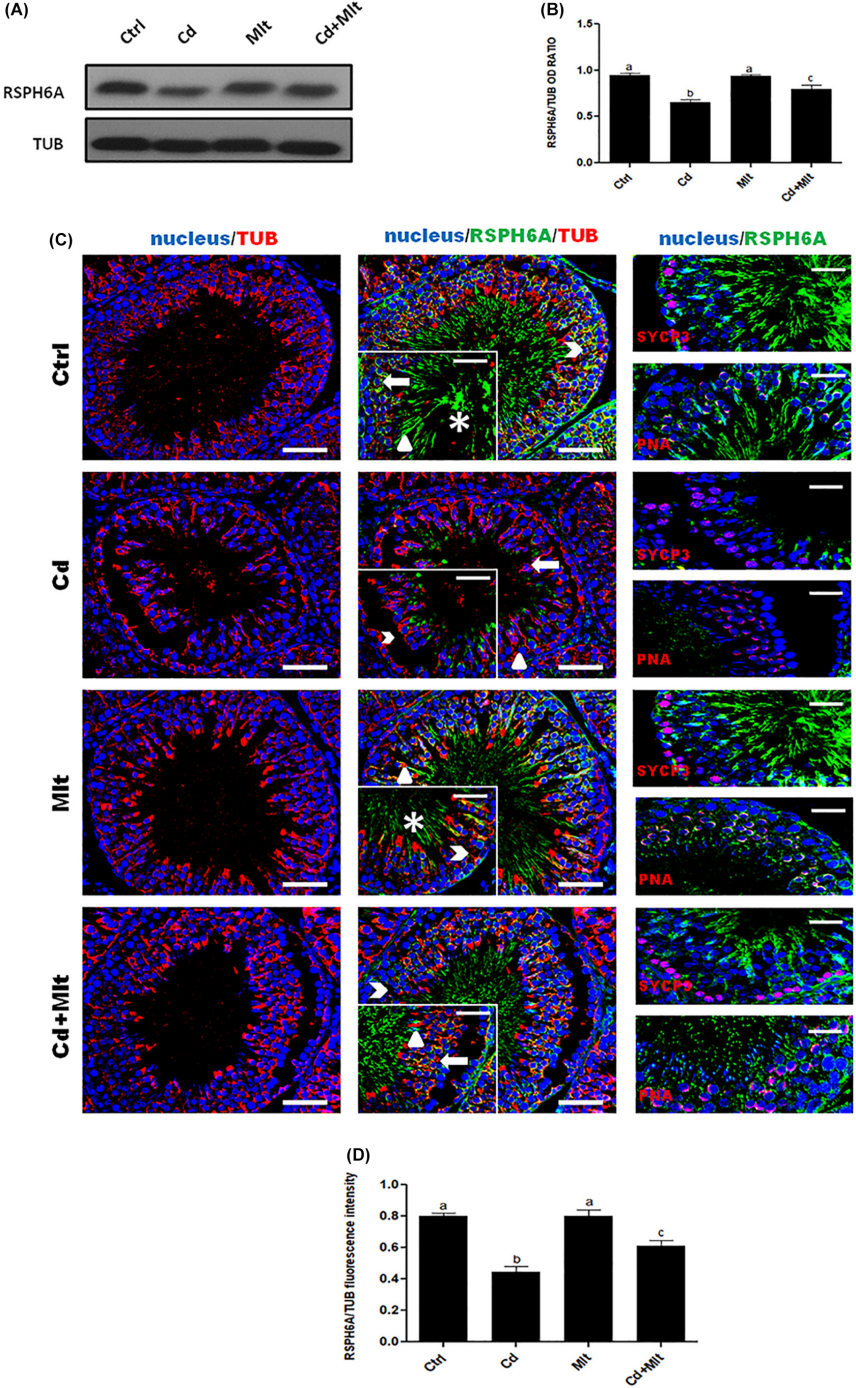
(A) WB and (B) relative histogram of RSPH6A in the testis of animals treated with Cd and/or Mlt. Data were normalized with α‐Tubulin and reported as OD ratio. a vs. b *p* < 0.001; b vs. c *p* < 0.05. (C) IF analysis of RSPH6A (green) and α‐Tubulin (red) in the testis of animals treated with Cd and/or Mlt. Cell nuclei were marked with DAPI (blue). For each group, the upper insets represent SYCP3 (red) and the lower insets represent PNA lectin (red). Arrowhead: SPC; thick arrow: RSpt; triangle: ESpt; asterisk: SPZ. Scale bars represent 20 μm. (D) Histogram showing the quantification of RSPH6A fluorescence signal intensity. a vs. b *p* < 0.001; b vs. c *p* < 0.01. All values are expressed as means ± SEM.

RPSH6A protein level was determined from the testis of rats treated with Cd and/or Mlt (Figure 3). WB analysis (Figure 3A,B) showed a significant decrease in RSPH6A levels in the Cd‐treated group when compared with Ctrl and Mlt groups (*p* < 0.001). In the Cd + Mlt group, RSPH6A level significantly increased compared with the Cd group (*p* < 0.05). No differences between Ctrl and Mlt‐treated groups were found. To confirm this data, we performed an IF analysis on testis from the four groups, together with the same markers described in section 3.1 (Figure 3C). The results were a consolidation of WB analysis; the Cd‐treated group showed a visible decrease in protein signal in the meiotic SPC, marked by SYCP3 (arrowhead), as well as in the post‐meiotic cells, highlighted by PNA lectin staining, RSpt (thick arrow), ESpt (triangle), and SPZ (asterisk) compared with the Ctrl and Mlt groups (*p* < 0.001), while the Cd + Mlt treated group showed a signal increase when compared to the Cd group (*p* < 0.01). There were no differences between Ctrl and Mlt‐treated groups (Figure 3D).

### 3.4 RSPH6A expression and localization in Cd and Mlt‐treated SPZ

**FIGURE 4:**
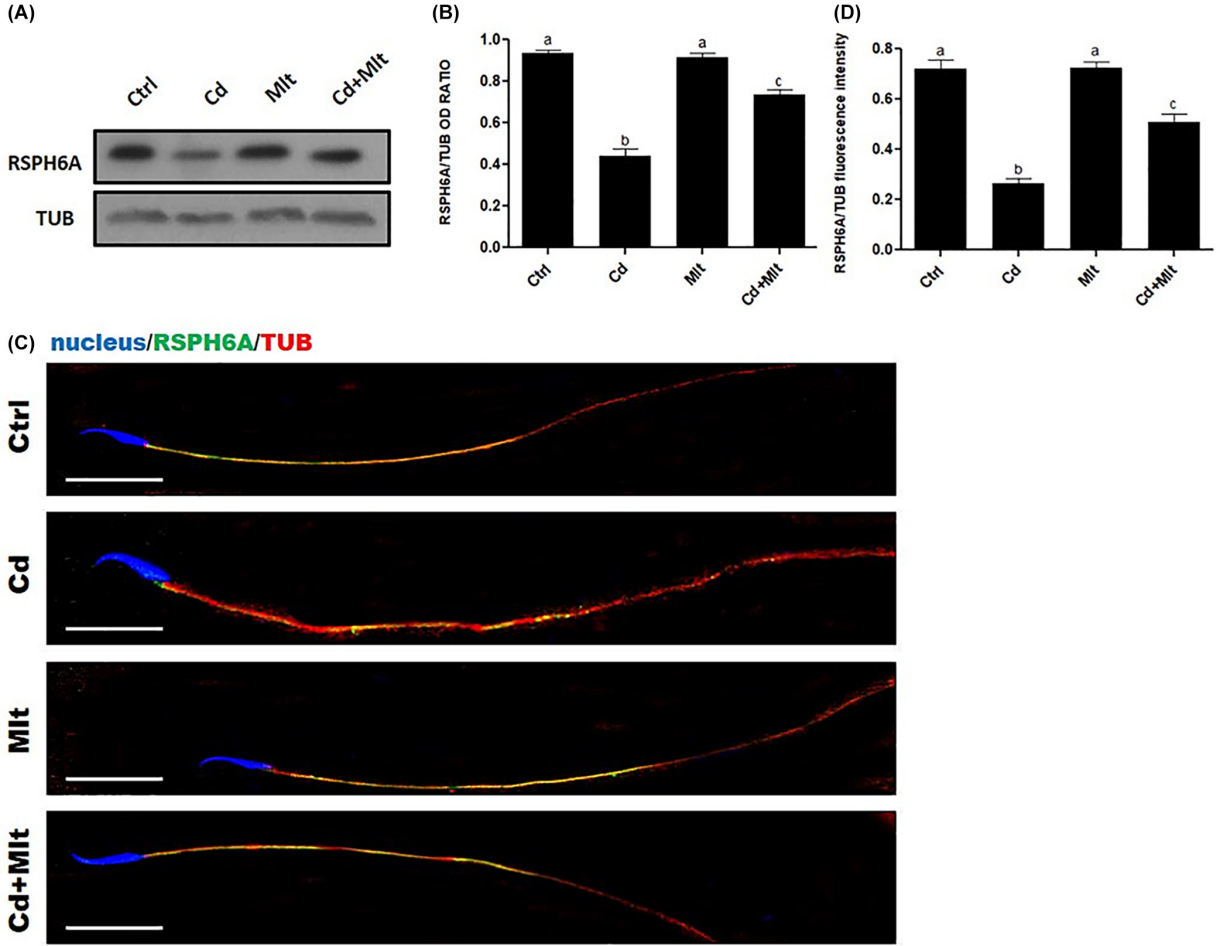
(A) WB and (B) relative histogram of RSPH6A in SPZ of animals treated with Cd and/or Mlt. Data were normalized with α‐Tubulin and reported as OD ratio. a vs. b *p* < 0.001; b vs. c *p* < 0.05. (C) IF analysis of RSPH6A (green) and α‐Tubulin (red) in SPZ of animals treated with Cd and/or Mlt. Cell nuclei were marked with DAPI (blue). Scale bars represent 20 μm. (D) Histogram showing the quantification of RSPH6A fluorescence signal intensity. a vs. b *p* < 0.01; b vs. c *p* < 0.01. All values are expressed as means ± SEM.

WB analysis on SPZ (Figure 4A,B) showed a significant decrease in RSPH6A expression in the Cd‐treated group when compared with Ctrl and Mlt groups (*p* < 0.001). Interestingly, when Mlt was administered along with Cd, there was a partial restoration of RSPH6A protein level, compared to Cd (*p* < 0.05), Ctrl and Mlt groups (*p* < 0.001; Figure 4A,B). To determine the localization of RSPH6A in the rat SPZ, we performed an IF on the SPZ of the four groups (Figure 4C). The results showed that the localization of RSPH6A is confined to the flagellum midpiece, as seen from the merge between α‐Tubulin and RSPH6A signals, in all the groups (Figure 4C). Finally, analysis of the fluorescence intensity showed a comparable pattern, statistically significant, as observed for the protein level (Figure 4D).

## 4 DISCUSSION

Successful reproduction, necessary to ensure the continuation of the species, requires the production of good‐quality gametes.^35^ In contrast, infertility is a principal concern today, affecting nearly 200 million people worldwide, of whom male infertility contributes 50 percent.^36^, ^37^, ^38^, ^39^ One of the most common diseases causing male infertility is asthenozoospermia, characterized by impaired motility of SPZ (<40%),^40^ which does not allow them to reach the oocyte, causing sub‐infertility.^39^ SPZ flagella formation occurs during spermiogenesis, an extremely dynamic process in which SPT intimately change their morphology. The whole spermatogenic process is regulated in a coordinated manner by several genes that are expressed in a spatial‐ and temporal‐specific pattern; however, the lack of an efficient in vitro system that mimics mammalian spermatogenesis encourages the use of other models, such as using knock‐out animals or studying the events occurring during the first wave of spermatogenesis.^41^ This last model is a helpful tool to characterize proteins involved in spermatogenesis and spermiogenesis due to the gradual appearance and differentiation of the cells composing the seminiferous epithelium. Thus, it becomes particularly crucial to study the cellular and molecular mechanisms underlying spermatogenesis to develop intervention strategies to enhance gametic quality and, therefore, to improve fertility.

In this context, our previous works confirmed the role of RSPH6A in human sperm motility, as its protein level and localization were impaired in abnormal conditions, such as DM1^6^ and induced oxidative stress.^8^ In this report, we analyzed, for the first time in rat testis, the temporal and spatial expression of RSPH6A during the first wave of spermatogenesis. WB analysis highlighted that the protein appeared at 21 PND, the developmental stage during which are produced I SPC, as well as a progressive increase in RSPH6A protein level until 60 PND. These results were confirmed by IF data, highlighting that the increased protein level, evidenced by WB, may be attributed to the gradual appearance of meiotic (SPC) and post‐meiotic (RSpt and ESpt) cells and, finally, mature luminal SPZ. We hypothesized that, although RSPH6A is necessary for the proper SPZ motility, its production during the stages preceding spermiogenesis, starting at 28 PND, may be needed to allow flagella formation. However, further studies are required to clarify this point. Nevertheless, our combined data confirmed the findings of previous works indicating the specific localization of RSPH6A in vertebrate GC.^2^, ^4^

Previously, we confirmed the involvement of RSPH6A in the regulation of sperm motility showing that *in vitro* Cd‐treatment of human SPZ, causing impairment of RSPH6A expression and localization, decreases their motility; simultaneous treatment with Mlt counteracted Cd effects.^8^ Therefore, we verified whether *in vivo* treatment with Cd affected rat RSPH6A protein in the rat testis. It is well known that the testis is particularly susceptible to the consequences of induced‐oxidative stress^42^; therefore, the choice of Cd was not accidental since its ability to generate oxidative stress and its effects on spermatogenesis have been widely demonstrated.^28^, ^43^ Thus, Cd treatment may be a useful model to study the effects of oxidative stress on spermatogenesis by unraveling the underlying molecular pathway(s).

In this report, we confirmed the detrimental effects caused by the oxidative stress Cd‐induced on testicular morphology, as well as the ability of Mlt to counteract these effects due to its well‐known antioxidant property.

As expected, results showed that 40 days of Cd treatment provoked a significant reduction in RSPH6A level in both the testis and SPZ. This data further confirms the detrimental action of Cd, which alters testicular activity at various levels, including steroidogenesis,^27^, ^43^ blood‐testis barrier integrity,^19^, ^21^ cytoarchitecture of the cells composing the seminiferous epithelium,^18^, ^25^, ^26^ and sperm parameters, especially motility.^8^, ^21^ Interestingly, the antioxidant ability of Mlt to counteract the Cd‐induced effect was evidenced again, as only a slight decrease in RSPH6A expression was observed in the group treated simultaneously with Mlt and Cd. Thus, the reduced SPZ motility induced by Cd may also be the consequence of RSPH6A expression impairment; this finding further supports its involvement in SPZ motility, also highlighted by the fact that Mlt, while preventing the decrease in RSPH6A levels, ensures proper SPZ motility.

In conclusion, this is the first report evidencing the expression and localization of RSPH6A in the rat testis and in mature SPZ. In particular, the protein localized in meiotic and post‐meiotic GC, starting at 21 PDN and accumulating during the following proliferative and differentiative stages occurring during the first wave of rat spermatogenesis. In addition, the involvement of RSPH6A in SPZ motility was partially blocked by Cd treatment and restored by the simultaneous use of the antioxidant Mlt. Finally, this report suggests that RSPH6A may be a potential new marker of male fertility but also confirms that Mlt is a helpful tool for improving or preserving SPZ quality.

## FUNDING INFORMATION

PRIN 2020 (prot. 20204YRYS5) and Erasmus KA‐1072020 (Naples‐Monastir).

## CONFLICT OF INTEREST STATEMENT

The authors declare no conflict of interest.
